# The use of ultrasonographic adrenal dimensions and the adrenal dimension-to-aorta ratio in the diagnosis of benign and malignant adrenal diseases in dogs

**DOI:** 10.14202/vetworld.2023.104-110

**Published:** 2023-01-12

**Authors:** Naparee Srisowanna, Chutimon Thanaboonnipat, Sirinun Pisamai, Kiatpichet Komin, Nan Choisunirachon

**Affiliations:** 1 Small Animal Hospital, Faculty of Veterinary Science, Chulalongkorn University, Henri-Dunant Road, Pathumwan, Pathumwan, Bangkok, 10330, Thailand; 2 Department of Veterinary Surgery, Faculty of Veterinary Science, Chulalongkorn University, Henri-Dunant Road, Pathumwan, Pathumwan, Bangkok, 10330, Thailand

**Keywords:** adrenal, aorta, dogs, hyperadrenocorticism, tumor, ultrasound

## Abstract

**Background and Aim:**

Ultrasound (US) is a useful tool for detecting adrenal abnormalities. However, a definite reference range differentiating normal and diseased adrenal glands in dogs of varying body sizes is still lacking. The organ dimension-to-aorta (Ao) ratio of the dogs is correlated with their body weight (BW). Therefore, this study aimed to investigate the adrenal dimensions, including adrenal pole thickness and adrenal length, as well as the adrenal dimension-to-Ao ratio, to differentiate between dogs with normal, benign lesions such as pituitary-dependent hyperadrenocorticism (PDH) and malignant invasive adrenal tumors.

**Materials and Methods:**

The medical records and US images of 39 dogs that were either normal (normal) (n = 15) or affected by PDH (n = 15) or malignant invasive adrenal tumors (tumor) (n = 9) were retrieved in this study. All the dogs had a transabdominal US on the sagittal plane. The adrenal dimensions and luminal Ao diameter at the peak of the systolic phase were recorded. The average adrenal dimensions, including the adrenal dimension-to-Ao ratio, were compared among the groups.

**Results:**

Most of the dogs in each group were small-breed dogs with comparable ages, BW, and Ao values. Both adrenal dimensions and the adrenal dimension-to-Ao ratio were significantly lower in the normal group than in the PDH and tumor groups. To differentiate the PDH group from the tumor group, adrenal dimensions of pole thickness and length were more appropriate than the adrenal dimension-to-Ao ratio.

**Conclusion:**

Adrenal dimensions and the adrenal dimension-to-Ao ratio can be used to diagnose adrenal diseases. However, in small-breed dogs, adrenal dimensions are suitable for differentiating PDH from tumor groups. Further research is required with a larger sample size and a wider range of canine body sizes.

## Introduction

Early diagnosis of adrenal disorders is essential for determining the severity and complications of these disorders, which include pituitary-dependent hyperadrenocorticism (PDH), a common adrenal disease in dogs, and adrenal tumor caused by abnormalities in the adrenal cortex or medullary parenchyma [1, 2]. While PDH in dogs is characterized by clinical manifestations, such as alopecia with chronic skin infection and high serum alkaline phosphatase levels [3], adrenal neoplasia is more malignant and can cause either contralateral adrenal hypoplasia in the case of adrenal cortical neoplasia or adjacent tissue invasion in the case of adrenal medullary neoplasia [1, 2]. The adrenal gland is typically located craniomedial to the kidneys, adjacent to the caudal vena cava on the right side and the aorta on the left side. Due to the normal variation, it has a more variable shape, particularly on the right side in dogs [4]. The maximal values of the adrenal size have been demonstrated in several reports. According to the first report, the average normal width of the left adrenal gland measured on the transverse plane in various dog breeds should not be more than 7.5 mm [5], whereas the cutoff value for normal adrenal dimensions in small-breed dogs is 6 mm [6]. It has been reported that normal adrenal dimensions range from 4 to 7 mm, whereas those of PDH range from 4 to 19 mm [7]. Despite the aforementioned data, the use of these values in veterinary clinical practice is difficult due to the difference in sizes among dog breeds. Therefore, the diagnostic procedure, which includes the clinical signs, routine laboratory workup, specific endocrine tests, and diagnostic imaging, is an important additional tool for differentiating adrenal lesions.

Ultrasound (US) is a useful imaging technique for assessing the adrenal glands because it is readily available, rapid, noninvasive, cost-effective, and highly sensitive and specific [2, 5, 6]. It can describe not only adrenal gland dimension, shape, margin, and echogenicity but also vascular or adjacent tissue involvement in cases with tumors. The normal size of the adrenal gland is an important factor in distinguishing adrenal hyperplasia and adrenal tumors from non-adrenal diseases [5–8]. The adrenal gland dimensions measured by the US have been widely reported. Although the upper limit of adrenal gland width, particularly at the pole, in healthy dogs has been addressed [5–8], dog breed and body weight (BW) are major factors that affect adrenal gland size [6, 9, 10]. This factor contributes to the ambiguous normal reference of the adrenal gland in dogs, leading to misinterpretation. Several reports have shown that organ dimension-to-aorta (Ao) ratios are useful for assessing normal organ sizes in dogs, including kidneys [11], caudal vena cava [12, 13], portal vein [14], and intra-abdominal lymph nodes [15], as well as the adrenal gland [16, 17]. However, the adrenal dimension-to-Ao ratio in the normal adrenal gland, PDH, and adrenal tumors has yet to be investigated.

This study aimed to compare the adrenal dimensions and the adrenal dimension-to-Ao ratio for differentiating normal (normal), PDH-affected, and malignant invasive adrenal tumor (tumor)-affected dogs. We hypothesized that the adrenal dimension-to-Ao ratio is better suited for differentiating adrenal diseases in dogs.

## Materials and Methods

### Ethical approval

All procedures involving retrospective data from animals in this study were approved by the hospital’s institutional review board, The Small Animal Hospital, Faculty of Veterinary Science, Chulalongkorn University (approval number: S151/2564.) All experiments were conducted in accordance with institutional guidelines and regulations, and the study followed the ARRIVE (Animal Research: Reporting of *in vivo* experiments) guidelines.

### Study period and location

The study included medical records and US images of dogs presented to the Small Animal Hospital, Faculty of Veterinary Science, Chulalongkorn University, from July 2018 to May 2021.

### Experimental design and animals

This study was conducted retrospectively. The dogs were divided into three groups: normal dogs (normal), PDH-affected dogs (PDH), and adrenal tumor-affected dogs (tumor). Data on PDH-affected and tumor-affected dogs were acquired from the hospital information system (HIS). The dogs were included in the PDH and tumor groups according to the following criteria: PDH: Dogs with a history of polyuria-polydipsia, external clinical signs of Cushing disease (alopecia, pendulous abdomen, and thin skin with comedones), a low dose dexamethasone suppression test (LDDST) indicating PDH (a cortisol level at 4 h <1.4 μg/dL, or <50% of basal cortisol level, and a cortisol level at 8 h >1.4 μg/dL) and evidence of bilateral, symmetrical enlargement of the adrenal glands on US images. Tumor: Dogs with evidence of adrenal gland enlargement and malignant behavior on US images, as indicated by adrenal invasion into adjacent anatomical areas such as vessels or muscles [2]. The normal group was then selected from the dogs with clinical demographic characteristics comparable to those of the PDH and tumor groups. These dogs were not affected by any adrenal-related or hypertensive diseases that may be affected by the adrenal size and Ao diameter. The dogs underwent US examination due to non-adrenal-related signs, and there were no clinical, hematological, biochemical, or adrenal US characteristics that indicated adrenal disease. Dogs with a history of long-term steroid administration, dogs with adrenal gland enlargement without laboratory diagnosis, dogs with previous medical treatment for PDH, and dogs with incomplete necessary information on US images, such as Ao diameter and adrenal dimensions, such as adrenal thicknesses at the cranial and the caudal poles of the adrenal gland, and adrenal length of both sides, were excluded from the study. Clinical and demographic data were then acquired from the HIS.

### Ultrasonography

**Figure-1:**
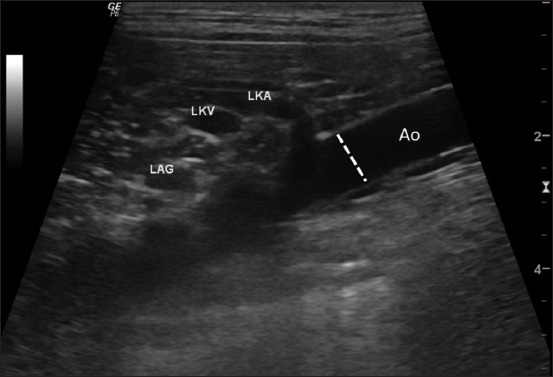
Ultrasonographic image of the mid-abdominal aorta. The internal aortic luminal diameter was measured at the mid-abdominal aorta caudally to the left renal artery root during the peak systolic phase (arrow).

All US images of dogs were performed by a Thai board-certified radiologist (NC) on the sagittal plane, using a 7–12 MHz linear or micro convex transducer (LOGIQ P6, General Electric, Korea) according to the dog’s body size. The US images were retrieved from the Picture Archiving and Communication System in a Digital Imaging and Communication System (DICOMs) format. All parameters, including the internal Ao luminal diameter at the caudal area of the root of the left renal artery during the systolic phase (Figure-1), maximal adrenal dimensions, including adrenal thicknesses at the cranial and the caudal poles of the adrenal gland, and adrenal length of the included dogs, were measured using digital calipers. In the case of an invasive adrenal tumor, the maximum thickness, including the invading area, was measured and recorded. Adrenal dimensions such as cranial pole thickness, caudal pole thickness, and adrenal length of the right and the left adrenal glands were compared between the normal and PDH groups. Furthermore, the average values of all adrenal dimensions and the adrenal dimension-to-Ao ratio from the left and right sides of the normal and PDH groups were compared to the tumor group.

### Statistical analysis

All statistical analyses were performed using GraphPad Prism 7.0 (GraphPad Software, San Diego, CA, USA). All clinical data for dogs in each group were presented as descriptive data, including mean, standard deviation (SD), median, and range. Before the comparison, all datasets were tested for normality using the Shapiro–Wilk normality test. The clinical and demographic data, Ao diameters, average adrenal dimensions, and averaged adrenal dimension-to-Ao ratio were compared between groups using the Kruskal–Wallis test, followed by Dunn’s post-hoc test. In addition, adrenal dimensions at the cranial and caudal poles, as well as length on either the right or left side, were compared between the normal and PDH groups using the Mann–Whitney U test, while Ao and BW were correlated using the *Spearman’s rank* correlation. p < 0.05 was considered statistically significant.

## Results

### Clinical and demographic characteristics

From July 2018 to May 2021, 15 and 9 dogs met the inclusion criteria for the PDH and tumor groups, respectively. The majority of the dogs, particularly those in the PDH and tumor groups, were small-breed dogs. In addition, 15 dogs that met the inclusion criteria for the normal group were selected. The clinical characteristics of the dogs in each group are shown in Table-1. In the normal group, there were five Chihuahua, three Shih tzus, and two Pomeranians, as well as one of each Pitbull, Yorkshire terrier, Poodle, Labrador Retriever, and Mixed breed. In the PDH groups, there were five Shih tzus, four Mixed breeds and three Chihuahuas, as well as one of each Poodle, Golden Retriever, and Pomeranian. In the tumor group, there were five Shih tzus and two Mixed breeds, as well as one of each type of Chihuahua and Dachshund. The ages and BW of the dogs did not differ significantly between the groups (p = 0.1818 for age and p = 0.3289 for BW, respectively).

**Table-1:** Clinical demographic information of normal dogs (normal), PDH and malignant, invasive adrenal tumor (tumor) affected dogs.

Parameters	Normal	PDH	Tumor
Number	15	15	9
Age (years)	10.6 ± 2.7 12.0 6–14	11.1 ± 4.3 11.0 3–17	12.8 ± 1.5 13.0 10–14
Body weight (kg)	8.3 ± 9.4 5.0 2.5–38.0	7.9 ± 8.4 6.0 1.6–36.5	7.9 ± 4 7.0 2.5–16.5
Sex			
Male			
All	6	6	7
Intact	2	3	5
Castrated	4	3	2
Female			
All	9	9	2
Intact	4	8	-
Spayed	5	1	2

All data of age and body weight in each group are presented with the mean value ± SD, median, minimum value and maximum value. SD=Standard deviation, PDH=Pituitary-dependent hyperadrenocorticism

### Ultrasonographic adrenal dimension

**Figure-2:**
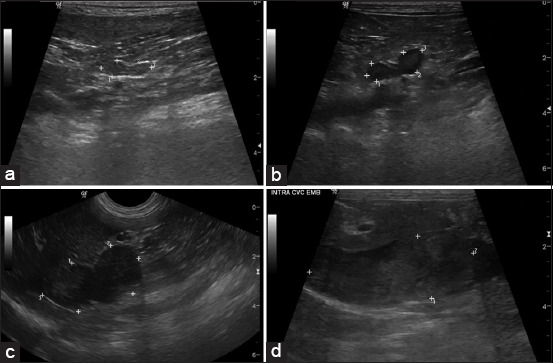
(a) Ultrasound images of the adrenal glands showing the appearances among normal adrenal gland, (b) pituitary-dependent hyperadrenocorticism, (c) malignant, invasive adrenal tumor that (d) includes the intravasation pattern.

In the US images, the adrenal glands in the normal group were elongated, oval to peanut-shaped, and hypoechoic (Figure-2a). The adrenal glands in the PDH group were slightly thicker, with a bulging contour and hypoechoic parenchyma (Figure-2b). In the tumor group, the adrenal glands varied in shape, contour, and echogenicity compared to the normal and PDH groups (Figures-2c and d). In the tumor group, four of nine dogs (44.4%) had unilateral enlargement with tumor invasion, whereas 5/9 dogs (55.6%) had bilateral enlargement with a unilateral tumor invasion. Tumor invasion was found in six (66.6%) and three (33.4%) dogs for the right and left adrenal glands, respectively. Most dogs (8/9, or 88.8%) had adrenal invasion into the cranial abdominal vena cava, whereas one dog (11.2%) had invasion into both the cranial abdominal vena cava and the epaxial muscle. The adrenal dimensions, including adrenal thickness at the cranial and caudal poles and adrenal length in each group, are shown in Tables-2 and 3. The average adrenal dimensions, including the average adrenal thickness from the cranial and the caudal poles and the adrenal length on the right and the left sides in the normal and PDH groups were compared to those of the ipsilateral malignant invasive adrenal tumor and the average adrenal thickness and length differed significantly between the groups (p < 0.0001). The average adrenal thickness was more appropriate for differentiating the normal versus PDH groups (p < 0.0001), the normal versus tumor groups (p < 0.0001), and the PDH versus tumor groups (p = 0.0028) than adrenal length (p = 0.0049, p <0.001, and p = 0.0408 for the normal versus PDH groups, normal versus tumor groups, and PDH versus tumor groups, respectively).

**Table-2:** The average adrenal dimension and average adrenal dimension-to-Ao ratio both of thickness and length in normal dogs (normal), PDH and malignant, invasive adrenal tumor (tumor) affected dogs.

Parameters	Normal	PDH	Tumor*
Pole thickness (cm)	0.46 ± 0.10^c^ 0.28–0.73	0.76 ± 0.30^a,c^ 0.33–1.82	2.90 ± 1.61^a,c^ 0.79–6.28
Length (cm)	1.72 ± 0.53^a,c^ 1.01–3.41	2.21 ± 0.59^a^ 1.06–3.46	3.31 ± 1.13^a,c^ 2.18–5.50
Pole thickness -to-Ao	0.80 ± 0.19^a,c^ 0.41–1.16	1.45 ± 0.77^a^ 0.62–4.09	1.60 ± 0.75^c^ 0.50–3.08
Length-to-Ao	2.87 ± 0.46^b,c^ 2.07–3.75	4.07 ± 1.17^b^ 2.46–8.02	5.89 ± 2.55^c^ 3.75–12.22

All data are presented with the mean value ± SD, minimum value and maximum value.

* Data represented only the ipsilateral side of the malignant, invasive adrenal tumor. The comparison of adrenal dimensions, each of average thickness from the cranial and the caudal poles and of length, among normal, PDH and Tumor, were done by the Kruskal–Wallis test, followed by Dunn’s post-hoc test.

a p < 0.05;

b p < 0.001;

c p < 0.0001. SD=Standard deviation, Ao=Aorta, PDH=Pituitary-dependent hyperadrenocorticism

**Table-3:** The adrenal dimension and adrenal dimension-to-Ao ratio between locations of the right and the left side in normal dogs (normal) and PDH-affected dogs.

Parameters	Normal		PDH	
	
	Right	Left	Right	Left
Cranial pole (cm)	0.48 ± 0.13^b^ 0.28–0.73	0.44 ± 0.08^c^ 0.32–0.57	0.95 ± 0.44^b^ 0.33–1.82	0.68 ± 0.16^c^ 0.43–1.03
Caudal pole (cm)	0.48 ± 0.11^b^ 0.29–0.63	0.48 ± 0.10^c^ 0.32–0.65	0.72 ± 0.33^b^ 0.34–1.71	0.70 ± 0.13^c^ 0.48–0.94
Length (cm)	1.75 ± 0.45 1.16–2.76	1.69 ± 0.58^a^ 1.01–3.41	2.17 ± 0.72 1.06–3.46	2.26 ± 0.45^a^ 1.76–3.36
Cranial pole-to-Ao	0.81 ± 0.16^c^ 0.52–1.12	0.77 ± 0.19^c^ 0.41–1.13	1.83 ± 1.11^c^ 0.75–4.01	1.27 ± 0.44^c^ 0.75–2.24
Caudal pole-to-Ao	0.82 ± 0.20 0.45–1.08	0.83 ± 0.21^c^ 0.46–1.17	1.39 ± 0.86 0.63–3.72	1.31 ± 0.38c 0.77–2.27
Length-to-Ao	2.95 ± 0.44 2.17–3.75	2.81 ± 0.46^c^ 2.08–3.59	3.99 ± 1.47 2.47–8.02	4.17 ± 0.83^c^ 2.88–5.76

All data are presented with the mean value ± SD, minimum value and maximum value. The comparison of adrenal dimensions, each of thickness at the cranial and the caudal poles and of length, between normal and PDH were done by the Mann–Whitney U test.

a p < 0.05;

b p < 0.001;

c p < 0.0001. SD=Standard deviation, Ao=Aorta, PDH=Pituitary-dependent hyperadrenocorticism

In terms of adrenal gland locations, the right adrenal gland showed a less significant difference in adrenal dimensions between the normal and PDH groups than the left adrenal gland (p = 0.0006, p = 0.0018 and p = 0.0699 for the cranial pole thickness, caudal pole thickness, and length of the right adrenal glands, respectively; and p < 0.0001, p = 0.0001 and p = 0.0019 for the cranial pole thickness, caudal pole thickness, and length of the left adrenal gland, respectively, as estimated using the Mann–Whitney U test).

### Ultrasonographic adrenal dimension-to-aorta ratio

The Ao diameters of dogs in the normal, PDH, and tumor groups were 0.61 ± 0.21, 0.56 ± 0.16, and 0.58 ± 0.16 cm, respectively. The Kruskal–Wallis test, followed by Dunn’s post-hoc test, revealed no significant difference in Ao diameters among groups (p = 0.792). In addition, Spearman’s rank correlation analysis revealed no significant correlation between Ao diameter and BW (p = 0.695). The adrenal dimension-to-Ao ratio, cranial pole thickness, caudal pole thickness, and adrenal length are shown in Tables-2 and 3. Similar to the adrenal dimension, the average adrenal dimension-to-Ao ratios, adrenal thickness, and adrenal length were significantly lower in the normal group compared to the PDH and tumor groups (p = 0.036 and p < 0.0001 for adrenal thickness in the normal vs. PDH groups and normal vs. tumor groups, respectively; p = 0.0004 and p < 0.0001 for adrenal length in the normal vs. PDH groups and normal vs. tumor groups, respectively). However, no significant differences in adrenal dimension-to-Ao ratios were found between the PDH and tumor groups (p = 0.077 and p = 0.172 for average adrenal thickness and length, respectively).

In terms of adrenal dimension-to-Ao ratio, the right adrenal gland also showed less significant differences in the adrenal dimension-to-Ao ratio between the normal and PDH groups than the left adrenal gland (p < 0.0001, p = 0.0215 and p = 0.0264 for the cranial pole thickness, the caudal pole thickness and the length of the right adrenal glands between groups; and p < 0.0001 for all of the cranial pole thickness, caudal pole thickness, and length of the left adrenal gland: Table-3).

## Discussion

Although clinical characteristics and laboratory investigations are the most commonly used for diagnosing adrenal disease, US is a useful, noninvasive, imaging modality for examining adrenal gland size, shape, and echogenicity [2, 5, 6, 18]. It is a common modality for diagnosing adrenal disorders, particularly in PDH, or adrenal tumors [2, 6, 7]. Adrenal tissue biopsy is the gold standard for classifying adrenal disorders [2, 19]. However, both the left and right adrenal glands are located near the aorta and the caudal vena cava, which are high-risk regions for fine-needle aspiration or tissue biopsy, especially in the case of non-sedative or non-anesthetic procedures. Thus, US images could provide more specific adrenal information, which allows for a less invasive diagnosis. One of the criteria for differentiating a normal adrenal gland from an enlarged one is its dimensions. However, it may not be suitable for determining adrenal lesions because it is influenced by the dogs’ breed, BW, age, and sex [6, 9, 10, 17, 18, 20]. Therefore, detecting adrenal disease requires an accurate interpretation of adrenal gland size by comparing the adrenal gland dimensions to Ao diameter. In this study, we investigated the effects of age, sex, BW, and Ao diameter on adrenal glands in three groups of dogs: normal, PDH, and tumor. Based on US images, the adrenal dimensions and the adrenal dimension-to-Ao ratio were examined. Our findings indicate that age, BW, and Ao diameter did not differ significantly among the three experimental groups, but sex distribution revealed a difference between the PDH and tumor groups. This might be due to the prevalence of PDH and tumors in small-breed dogs. There were significant differences in adrenal dimensions between the dog groups of the normal and PDH group in term of the cranial pole, caudal pole, and adrenal length and the adrenal dimension-to-Ao ratio differed significantly between the normal and PDH groups but not between the PDH and tumor groups. This finding may be useful in selecting appropriate techniques to characterize adrenal lesions in veterinary practice.

To obtain comparable data across groups, medium-to-old-aged, normal small-breed dogs were selected for the normal group. Although all dogs in the PDH group were confirmed based on serum cortisol levels in the LDDST in conjunction with abdominal US results, nine dogs in the tumor group had no LDDST results. All dogs in the tumor group had an aggressive adrenal mass on abdominal US examination. According to a previous study, aggressive adrenal behavior indicates a malignant adrenal tumor [2]. Therefore, these dogs were categorized into the tumor group; however, no dogs had laboratory or histopathological findings indicating malignant tumors.

Although a previous study found that Poodles, Dachshunds, and miniature Schnauzers are more prone to adrenal diseases [21], Shih Tzus were the most affected by adrenal diseases in both the PDH and the tumor groups in our study. In a recent study, PDH was also found to be more prevalent in females than in males [22]. In contrast, tumor-affected dogs were mostly male [1, 19]. In our study, the adrenal disease was common in medium- and old-aged dogs, with slightly higher incidences among breeds and between sexes, which may be due to the small number of dogs in the study or the different geographic regions. Shih Tzu, for example, is one of the most popular breeds in Thailand.

Although some previous reports have shown that the adrenal gland can be normal in size and shape, the US images of PDH-affected dogs in the current study mostly showed bilateral symmetrical adrenal enlargement [7, 18]. In this study, the invasive adrenal tumor-affected blood vessels, such as the caudal vena cava as well as local adjacent soft tissue structures, such as the epaxial muscle. According to the previous study [2], the adrenal tumor with vascular invasion affected the right side more significantly than the left side. Although adrenal metastasis is rare, the abdominal caudal vena cava and the abdominal aorta are frequently invaded by adrenal malignant tumors, particularly adrenocortical carcinoma [23]. Thus, vascular invasion and tumor thrombosis can be used to aid in the diagnosis of malignant adrenal tumors.

As previously described, adrenal length and height measured on the sagittal plane were significantly correlated with BW and showed less variation, particularly in small-breed dogs [9, 16, 17]. Moreover, the caudal pole of adrenal thickness measured on the sagittal plane was significantly correlated with gross appearance [24]. Hence, the adrenal gland dimension and intra-luminal Ao diameter were only assessed using sagittal US images. As expected, our findings revealed that average adrenal thickness and length differed significantly among the three groups. However, adrenal thickness outperformed adrenal length in differentiating between the normal and PDH groups. Furthermore, the dimensions of the left adrenal gland differed significantly from those of the right. The lower significance of adrenal dimensions between the normal and PDH groups on the right side may be due to the difficult examination process [5, 6, 18, 25].

Recent studies have reported the adrenal dimension-Ao ratio in dogs using the abdominal US. In dogs, BW affected both the Ao diameter and the adrenal dimension-Ao ratio [16, 17]. However, those reports were based on healthy dog groups. Our study is the first to show differences in the adrenal dimension-to-Ao ratio among normal, PDH, and Tumor groups. Interestingly, the adrenal dimension-to-Ao ratio was less suitable for differentiating between adrenal characteristics, particularly between the PDH and tumor groups, than the adrenal dimensions. This may be due to the fact that most dogs in this study were small-breed dogs with less variable body size and no relationship between intra-luminal Ao diameter and BW. Therefore, as adrenal diseases are more common in small-breed dogs, the adrenal dimension may be more appropriate than the adrenal dimension-to-Ao ratio.

## Limitations of the study

Our study has some limitations. First, it is a retrospective study, and the diagnosis methods in all groups did not follow the same protocols. Specific endocrine tests, such as LDDST, were not performed on the normal and tumor groups. The PDH group was formed based on the LDDST results, but these findings were not confirmed by computer tomography or magnetic resonance imaging. The dogs in the normal and tumor groups were assigned based on their history, physical examination, routine blood analysis, and abdominal US. Therefore, we could not be certain that asymptomatic hypoadrenocorticism was excluded and occult PDH was included. Furthermore, the results of tissue sampling were not shown for all dogs due to the conservative and medical treatments. Second, the dominant dog breeds in this study would be influenced by the different styles of pet owners across geographic locations. Third, because this was a retrospective study, there were a small number of dogs in each group, and the inter- and intra-observer variability was not accessible.

## Conclusion

Our findings suggest that the US evaluation of adrenal dimensions and adrenal dimension-to-Ao is a useful parameter for distinguishing adrenal from non-adrenal disease. However, the adrenal dimension-to-Ao ratio was inadequate to differentiate PDH from tumor groups, particularly in small-breed dogs. More research with a larger sample size and a wider range of body size in adrenal disease-affected dogs may yield more interesting results.

## Authors’ Contributions

NS and NC: Study conception and design. NS and NC: Acquisition of data. NS, CT, SP, KK, and NC: Analysis and interpretation of data. NS, CT, SP, and NC: Drafting of manuscript. NS, CT, SP, KK, and NC: Critical revision. All authors have read and approved the final manuscript.
